# Seroprevalence and Factors Associated with *Toxoplasma gondii*, *Neospora caninum*, and *Besnoitia besnoiti* Infections in Cattle and Goats in Selangor, Malaysia

**DOI:** 10.3390/ani13050948

**Published:** 2023-03-06

**Authors:** Mohammed Babatunde Sadiq, Azim Salahuddin Muhamad, Siti Aisyah Hamdan, Siti Zubaidah Ramanoon, Zunita Zakaria, Nor Azlina Abdul Aziz, Rozaihan Mansor, Siti Suri Arshad, Nurulhidayah Khalid, Norhamizah Abdul Hamid, Juriah Kamaludeen, Sharifah Salmah Syed-Hussain

**Affiliations:** 1 Department of Farm and Exotic Animal Medicine and Surgery, Faculty of Veterinary Medicine, Universiti Putra Malaysia, Serdang 43400, Selangor, Malaysia; 2 Department of Veterinary Clinical Studies, Faculty of Veterinary Medicine, Universiti Putra Malaysia, Serdang 43400, Selangor, Malaysia; 3 Department of Veterinary Pathology and Microbiology, Faculty of Veterinary Medicine, Universiti Putra Malaysia, Serdang 43400, Selangor, Malaysia; 4 Department of Animal Science and Fisheries, Faculty of Agriculture and Food Science, Universiti Putra Malaysia Bintulu, Sarawak Campus, Bintulu 97008, Sarawak, Malaysia

**Keywords:** seroprevalence, *Toxoplasma gondii*, *Besnoiti besnoiti*, *Neospora caninum*, cattle, goats, Malaysia

## Abstract

**Simple Summary:**

*Toxoplasma gondii*, *Neospora caninum*, and *Besnoitia besnoiti* are important causes of production losses in small and large ruminants. These parasites could result in severe economic losses to farmers if they are not effectively controlled. The prevalence of these parasites in smallholder farms in Malaysia is underreported. Thus, this study attempted to determine the level of exposure of cattle and goat population from 19 farms in Selangor, Malaysia to *T. gondii*, *N. caninum*, and *B. besnoiti* using ELISA test kits. Results revealed that the prevalence of *T. gondii*, *N. caninum*, and *B. besnoiti* antibodies in the sampled cattle was 5.3%, 2.5%, and 5.7%, respectively. The corresponding seroprevalence in the sampled goats was 69.8% for *T. gondii* and 3.9% for *N. caninum antibodies*. Further analyses demonstrated that older animals, semi-intensive management systems, the presence of dogs or cats on farms, a large herd size (>100 animals), and the source of replacement stock increased the risk of exposure to *T. gondii*. These findings reflect the extent of *T. gondii*, *N. caninum*, and *B. besnoiti* infections in smallholder farms in Selangor. More research is required to develop effective measures to control these important parasites at the national level.

**Abstract:**

Apicomplexan parasites such as *Toxoplasma gondii*, *Neospora caninum*, and *Besnoitia besnoiti* are widely recognized as causes of production diseases in ruminants. This study aimed to investigate the serological occurrence of *T. gondii*, *N. caninum*, and *B. besnoiti* in cattle and goats from smallholder farms in Selangor, Malaysia. A cross-sectional study was conducted on 19 farms by collecting 404 bovine (n = 225) and caprine (n = 179) serum samples, which were then essayed for *T. gondii*, *N. caninum*, and *B. besnoiti* antibodies using commercially available ELISA test kits. Farm data and animal characteristics were documented, and the data were analyzed using descriptive statistics and logistic regression models. The seroprevalence of *T. gondii* at animal and farm levels in cattle was 5.3% (95% CI 1.2–7.4%) and 36.8% (95% CI 22.4–58.0%), respectively. Animal-level seropositivity for *N. caninum* was 2.7% (95% CI 0.4–4.2%) and 5.7% for *B. besnoiti* (95% CI 1.3–9.4%) with corresponding farm-level seropositivity of 21.0% and 31.5%, respectively. For the goat samples, a high animal- (69.8%; 95% CI 34.1–82.0%) and farm-level (92.3%) seropositivity was recorded for *T. gondii*, but was relatively lower for *N. caninum* antibodies, at 3.9% (95% CI 1.5–6.2%) and 38.4% (5/13). The factors associated with *T. gondii* seropositivity were older animals (above 12 months) (OR = 5.3; 95% CI 1.7–16.6), semi-intensive farms (OR = 2.2; 95% CI 1.3–6.2), the presence of either dogs or cats (OR = 3.6; 95% CI 1.1–12.3), a large herd size (>100 animals) (OR = 3.7; 95% CI 1.4–10.0), and a single source of replacement animals (OR = 3.9; 95% CI 1.6–9.6). These findings are vital in developing effective control measures against these parasites in ruminant farms in Selangor, Malaysia. More national epidemiological research is required to elucidate the spatial distribution of these infections and their potential impact on Malaysia’s livestock industry.

## 1. Introduction

Apicomplexa protozoan parasites, *Toxoplasma gondii*, *Neospora caninum*, and *Besnoitia besnoiti*, have a common heterogeneous life cycle and contribute significantly to the reproductive failures and low efficiency in ruminant farms globally [1,2,3]. *Toxoplasma gondii* infects all warm-blooded animals and causes toxoplasmosis, a cosmopolitan zoonotic disease that is of public health significance in humans, especially in immunocompromised individuals and unborn fetuses. Human toxoplasmosis causes several health issues including fever, dizziness, arthralgia, fever, myalgia, and lymphadenopathy, whereas abortions, blindness, and encephalitis may occur in severe conditions [4].

Several routes of *T. gondii* infection exist in humans, with the most common route being via the consumption of food products of animal origin [4]. Small ruminants’ meat contains a high number of tissue cysts, which is linked to grazing in environments contaminated with oocysts [2]. Hence, a major source of infection stems from poor food hygiene practices and/or undercooking of meat, especially in countries where the consumption of sheep and goat meat is part of the culinary tradition [5]. High rates of *T. gondii* infection in small and large ruminants results in severe economic losses and imposes significant zoonotic health hazards among susceptible populations [6]. The wide availability of the definitive (domestic cats and other wild felids) and intermediate (mammals and birds) hosts contribute to the high infection rates of *T. gondii* and variation in the prevalence in different countries [4].

*Neospora caninum* is also a major concern in large and small ruminants. The protozoan parasite is widely recognized as a major abortifacient pathogen in cattle but has also been shown to cause abortion in goats and sheep [7]. Additionally, the pathogen is common in these ruminant species with varying seroprevalence values, which are strongly associated with breeding conditions [1,7]. Previous studies have shown that extensive management systems, herd size, and pasture access are common risk factors for neosporosis in small ruminants [5,8].

Bovine besnoitiosis contributes to economic losses in affected farms due to skin lesions, weight loss, prolonged recovery, complete or transient sterility in males, and reduced milk production [9]. Typical cases of bovine besnoitiosis transit from acute to chronic stages with varying pathogenicity and abortion might occur in infected cows [10]. Overall, the clinical impact and epidemiology of *B. besnoiti* on ruminants are not fully understood. Nevertheless, their capacity to induce abortion and general disorders in the domestic intermediate host have been documented [11]. For instance, bovine besnoitiosis was recognized as an emerging disease in European countries following the reported cases in Germany [12], Spain [13], and Italy [14]. Overall, several researchers concluded that limited information is available on the epidemiology, prevalence, and clinical evidence of besnoitiosis infections in ruminants [2,11].

The rearing of cattle and goats are key activity by Malaysian smallholder farmers, thus reflecting the importance of ruminants in income generation for several livelihoods in the country. A recent study conducted in Selangor, Malaysia, highlighted the importance of *T. gondii* infection in meat samples collected from wet markets and abattoirs in the region [15]. Meanwhile, a seroprevalence survey has also documented the potential definitive hosts of the parasite in Selangor [16]. On the other hand, Kyaw et al. [17] reported the first seropositivity of *Neospora* in goats and sheep in Malaysia. The study was undertaken by sampling 472 animals from 10 districts of Kelantan, and the resulting prevalence of *Neospora* antibodies was 1.1%. Other local studies by Cheah [18] and Rahman et al. [19] focused on calf and cattle, respectively. Thus, epidemiological data about *T. gondii* and *Neospora*, especially in the ruminant population, remain scarce in Malaysia. Presently, there is a dearth of information regarding the exposure of ruminant farms to *N. caninum* and *B. besnoiti* in the country. Seroprevalence and risk factors information of the aforementioned protozoan parasites is important in developing and implementing future public health programs to control their infections in Malaysian ruminant farms. Hence, this study aimed to determine the serological occurrence of *T. gondii*, *N. caninum*, and *B. besnoiti* in cattle and goats from smallholder farms in Selangor, Malaysia. The secondary goal was to identify the factors associated with the seropositivity of each parasite in the study location.

## 2. Materials and Methods

### 2.1. Study Design, Study Area, and Sampling Technique

A cross-sectional study was conducted involving ruminant farms in Klang Valley, Selangor, Malaysia. As of 2018, there were 122 registered cattle and goat farms with the Department of Veterinary Services (DVS), Selangor. The farmers were contacted either via phone contacts or email addresses and informed about the purpose of the study while seeking their oral consent to participate. Farmers willing to participate were briefed about the inclusion criteria, which entailed the production of either cattle or goats for commercial or subsistence purposes, herd size > 10 animals, location within any district in Selangor, and being free from any reportable diseases as at the proposed time of farm visit.

The required sample size was calculated using the EpiTools website (http://epitools.ausvet.com.au) (accessed on 4 August 2019) by assuming an expected seroprevalence of 35.5% from a previous study conducted in Malaysia by Chandrawathani et al. [20] at a confidence level (CI) of 95%, precision level of 5%, and a target population of ruminants (cattle, goat, and sheep) in Selangor as 40,000 animals [21]. The minimum required sample size based on the aforementioned parameters was 350.

### 2.2. Collection of Farm and Animal Data

A well-structured datasheet was used to record vital on-farm data such as herd size, farm location, management systems, pest control, presence of stray animals (cats, dogs, wild animals, etc.), feeding regimen, water source, and the source of animals. Meanwhile, animal characteristics such as sex, age, breed, and identification number were recorded. All the information was provided by the farm owner or available farm staff during the researcher’s visit.

### 2.3. Blood Sampling and Serology

All the animals were restrained effectively for blood sampling via the jugular vein. The samples were transported immediately to the laboratory for further analysis. Of the 22 farms that were visited in this study, a total of 404 serum samples were collected, comprising 225 from cattle and 179 from goats. The samples were centrifuged at 5000 rpm to obtain the sera, transferred into 1.5 mL microcentrifuge tubes, and stored frozen at −80 °C prior to essay for *T. gondii*, *N. caninum*, and *B. besnoiti* antibodies. This study was approved by the Institutional Animal Care and Use Committee University Putra Malaysia (IACUC) (Approval code: U066/2018).

#### 2.3.1. ELISA Testing for Specific *T. gondii* Antibodies

All sera collected were tested using a commercially available ELISA test kit (ID Screen^®^ Toxoplasmosis Indirect Multi-Species Test Kit, ID.vet, France). This indirect assay utilizes *T. gondii* P30 antigen with a multi-species peroxidase (HRP) conjugate and detects antibodies against *T. gondii* antibodies in samples from cats, dogs, swine, and ruminants and plates were read at 450 nm. For interpretation of the results, the sample to positive (S/P) percentage was calculated as: S/P % = (ODsample − ODNC)/(ODPC − ODNC) × 100. Any sample with an S/P % less than or equal to 40% was considered negative, a value greater or equal to 50% was considered positive, and between 40–50% was considered doubtful. According to the manufacturer, this cut-off has more than 99% specificity and 97% sensitivity. The test was considered valid if the mean value of the positive control OD (ODPC) is greater than 0.350 (ODPC > 0.350) and the ratio of the mean OD values of the positive and negative controls (ODPC and ODNC) is greater than 3 (ODPC/ODNC > 3).

#### 2.3.2. ELISA Testing for Specific *N. caninum* Antibodies

All samples were also analyzed for the presence of *N. caninum* antibodies using a commercial competitive ELISA kit (cELISA- VMRD, VMRD, Pullman, DC, USA). According to the manufacturer’s instructions and cut-off recommendations, serum samples were considered positive when the inhibition percentage was greater than 30%. Farms were considered positive when their true seroprevalence was greater than 0 as described by von Blumröder et al. [22].

#### 2.3.3. ELISA Testing for Specific *B. besnoiti* Antibodies

Only cattle sera were tested for *B. besnoiti* using a commercially available ELISA test kit (ID Screen^®^ Besnoitia Indirect, IDvet, France). The procedure was based on the manufacturer’s instructions. Briefly, the test kit was specific for cattle only and coated with *B. besnoiti* purified antigen. The conjugate used was anti-ruminant IgG-HRP conjugate and the assay was read using a spectrophotometer with a wavelength of 450 nm. This assay has a 97.2% sensitivity and 100% specificity [23] and the result was interpreted and calculated using the formula below.

S/P % = (Net OD sample/Net OD positive control) × 100

Sample to Positive (S/P) values of less than or equal to 25% were considered negative, whereas those greater than or equal to 30% were considered positive. Meanwhile, S/P values less than 30% but greater than 25% were considered doubtful and recorded as negative in this study.

### 2.4. Data Analysis 

All farm and animal data were manually transferred from the datasheet to the Microsoft Excel spreadsheet (NY, United States). Upon completing the sampling, the data were sorted and checked for any entry errors before transferring to SPSS Version 23 (IBM, Armonk, NY: IBM Corp, the United States) for statistical analysis. Descriptive statistics were employed to summarize the farm and animal data, thereby presenting the farm and animal characteristics. Continuous variables were assessed for data normality using the level of kurtosis and skewness. The data were then presented either as means (±SD) or medians (±IQR) depending on the normality test results. All categorical data were presented in frequencies and percentages.

Seropositivity was computed based on the number of seropositive animals to the total number of animals under investigation for each apicomplexan protozoan. A mixed-effects logistic regression model was only built separately to determine the factors associated with seropositivity for *T. gondii* in cattle and goat populations, as the seroprevalence of *N. caninum* and *B. besnoiti* was low to accommodate risk factor analysis. Seropositivity to *T. gondii* at animal levels was considered the dependent variable and the farm was introduced into the model as a random factor. All other independent variables with complete data records were introduced into the model in a backward conditional method. Factors such as water source and feeding regimen were not considered since a significant number of farms failed to provide the information. Variables were removed from the model upon assessing the Akaike information criterion (AIC) before obtaining the final model fit. All biologically plausible pair-wise interactions were evaluated.

## 3. Results

### 3.1. Descriptive Analysis

Table 1 shows the characteristics of the study population and the corresponding seroprevalence towards each apicomplexan. Overall, a higher proportion (42.8%) of the sampled animals were between 13 and 24 months old and female (89.0%). Most of the animals (82.1%) were reared under an intensive management system and not exposed to pest control (79.4%). Other farms and animal characteristics such as herd size, presence of stray animals, source of animals, type of production, and water source are presented in Table 1.

### 3.2. Seroprevalence at Animal and Farm Levels

As shown in Table 2, the individual seroprevalence of *T. gondii* in cattle was low (5.3%; 12/225), while seven farms (7/19; 36.8%) were seropositive for the parasite. Likewise, a low number of cattle tested positive for *N. caninum* (2.7%; 6/225) and *B. besnoiti* (5.7%; 13/225) with a corresponding number of seropositive farms of four (21.0%) and six (31.5%), respectively. Only a single farm (Farm 5; F5) was seropositive for *T. gondii* and *N. caninum* involving only one animal. Meanwhile, two farms (F5 and F13) were seropositive for *N. caninum* and *B. besnoiti*, involving three animals. Interestingly, Farm 5 was also seropositive for the three apicomplexan parasites: *T. gondii*, *N. caninum*, and *B. besnoiti.*

Table 3 depicts the seroprevalence of *T. gondii* and *N. caninum* in the goats sampled from 13 farms. High seropositivity was recorded for *T. gondii* at 69.8% (125/179) at the animal level, with a corresponding farm-level seroprevalence of 92.3%, as only one farm (F8) had no seropositive animal. Meanwhile, the seroprevalence for *N. caninum* antibodies was low at 3.9% (7/179) but five of the farms (38.4%; 5/13) had at least one seropositive animal. All the farms that were seropositive for *N. caninum* had at least one animal seropositive for *T. gondii.*

### 3.3. Factors Associated with Seropositivity for T. gondii, N. caninum, and B. besnoiti

Table 4 presents the final mixed-effects logistic regression model for the factors associated with the seroprevalence of *T. gondii* in goats. The factors associated with *T. gondii* seropositivity included age, management system, presence of stray animals, animal source, and herd size (Table 5). Older goats (above 12 months) were more likely to be seropositive (OR = 5.26; 95% CI 1.72–16.66) compared to younger ones (less than 6 months). Likewise, there were higher odds of seropositivity in semi-intensively kept goats (OR = 2.17; 95% CI 1.34–6.21) relative to those raised intensively, as well as in farms with the presence of either dogs or cats (OR = 3.63; 95% CI 1.1–12.3) relative to those without such animals. Farms with fewer animals (less than 50) were less likely to be seropositive (OR = 0.27; 95% CI 0.10–0.72) compared to those with more than 100 animals. Farms using a single source or own animals for replacement had higher seroprevalence (OR = 3.92; 95% CI 1.61–9.69) compared to farms with multiple or external sources.

For cattle, only the management system was significantly associated with seropositivity for *T. gondii* antibodies (Table 5). Cattle raised under the intensive system were less likely to be seropositive (OR = 0.20; 95% CI 0.06–0.69) compared to those raised under semi-intensive systems. Meanwhile, the presence of stray animals (i.e., dogs, cats, wild animals) and pest control were not significant in the final models. Given the low seroprevalence of *N. caninum* and *B. besnoiti* in both cattle and goats, risk factor analysis was not conducted.

## 4. Discussion

This study was the first attempt to investigate the seroprevalence of the three most common apicomplexan protozoa, *T. gondii*, *N. caninum*, and *B. besnoiti*, in bovine and caprine livestock in Malaysia. The results revealed a low exposure of cattle farms to *T. gondii* as both the animal-level seroprevalence was 5.3% while only 7 of the 19 farms had at least one seropositive cattle. This result aligns with a previous conducted in the same study area in Malaysia where 9.0% of sampled cattle were seropositive for *T. gondii* antibodies [18]. In comparison to studies conducted elsewhere, the estimate from the cattle population in this study differs from pooled seroprevalence of 28.5% in Asia, 18.8% in Africa, 21.9% in Europe, 22.2% in America, and 1.36% in Australia/Oceania, as reviewed by Shariatzadeh et al. [24]. Direct comparisons between studies conducted in different countries might not be feasible due to variations in study designs, the serological tests employed, cut-off values used, geographic locations, and farm practices. These events might explain the discrepancies in seropositivity rates reported in different studies. Nevertheless, the majority of seroprevalence studies employed a cross-sectional design.

In this study, a relatively higher seroprevalence of *T. gondii* was detected in goats (69.8%), with 12 of the 13 farms having at least one seropositive animal. These findings are similar to previous reports in goat and sheep samples collected from wet markets in Selangor in which 69.0% and 35.0% were seropositive for *T. gondii* [15]. Likewise, a high prevalence of *T. gondii* antibodies was reported in goat populations from Egypt (62.0%), Italy (63.3%), Iran (48.0%), and Pakistan (42.8%) [25,26,27], while relatively lower estimates were found in Myanmar (11.4%) [28]. Factors contributing to the varying exposure to the parasite, the serological assay and cut-off values used, and contamination at farm levels might explain the various seroprevalences detected in the aforementioned studies. The similarity in the seroprevalence found in the present study and earlier research conducted on wet market samples suggest consistent levels of exposure to *T. gondii* and environmental contamination by the parasite in various ruminant farms in Selangor. Nevertheless, given that the present study included both young and adult goats, the seroprevalence of *T. gondii* is alarming since the previous study involved adult animals slaughtered at abattoirs, which are presumably at a higher risk of infection [29]. Thus, the high seroprevalence of *T. gondii* detected in this study implies a significant public health risk.

This study supports previous research findings that small ruminants (goats and sheep) are more susceptible to *T. gondii* infection compared to large ruminants such as cattle and buffaloes [2,3]. Accordingly, 69.3% of sampled goats were seropositive compared to 5.3% of sampled cattle from various farms in Selangor. The lower infection rate in the latter might be due to greater resistance and a stronger immune system compared to that obtainable in goats [2].

*Besnoitia besnoiti* and *N. caninum* have become important pathogens in cattle and goat farms, causing severe economic losses in endemic farms worldwide. A low number of cattle were seropositive for *N. caninum* (2.7%; 6/225) and *B. besnoiti* (5.7%; 13/225) in this study. Most studies conducted elsewhere have reported varying seroprevalences for *B. besnoiti* in cattle populations, including 2.7% in Turkey [30] and Australia [31], as was found in the present study, and are comparatively lower than the 22.0% reported in Greece [32] and 44.1% in Italy [33]. Although there is no report of bovine besnoitiosis in Malaysia, evidence of the spread of the disease was documented in other nations such as Germany, Italy, and Portugal following subsequent outbreak reports (reviewed by Jacquiet et al. [34]. In the current study, four farms had a history of importation of cattle mainly from Australia and Thailand. Some studies reported that the importation of cattle from endemic countries such as France and Spain increased the risk of bovine besnoitiosis [35]. For instance, an outbreak of besnoitiosis occurred in a beef cattle herd in Bavaria [35] while positive animals were discovered in Italy following the importation of cattle from France [33]. These findings indicated that cattle from endemic regions were able to cause transmission of the disease to a naive region.

One reason for the geographical expansion of the besnoitiosis is the trade between countries. Presently, Australia is one of the main countries from where Malaysian farmers source their cattle [36]. Though Malaysia does not share direct animal trade with reported endemic countries, seropositive findings among cattle farms in Australia were linked to trade with other European nations. Previous studies conducted in Australia also reflected a higher presence of antibodies against *B. besnoiti* in cattle sampled from different parts of the country [37]. Hence, the importation of cattle from endemic regions into Malaysia could be a risk factor and requires further investigation.

*B. besnoiti* are transmitted either by direct contact with oocysts or by mechanical transmission via vectors. Fly control is important as flies play a role as the vector for the parasite. In the current study, six farms practiced the burning of coconut husks to produce smoke as their method of controlling the number of flies, especially in the morning. The transmission of *B. besnoiti* from cattle to cattle might occur through mechanical transmission by blood-sucking flies, and *B. besnoiti* DNA had been detected in stable flies after ingestion of blood from the infected cattle [38]. In Malaysia, biting flies such as stable and Tabanidae flies have been reported [39]. Though further investigation is required, farmers must be made aware of the importance of hygiene and fly control on their farms.

The overall seroprevalence of *N. caninum* in the present study (2.7%; 6/225) is slightly higher than the report (0.32%) by Kyaw et al. [40] among sheep and goats in Musang district in Kelantan, Malaysia, but is similar to the earlier study conducted by the same group of researchers, which was 1.1% [17]. These seroprevalence estimates are generally lower than the pooled estimate of 6.2% recorded by Reichel et al. [41] in a detailed systematic review and meta-analysis. We also found that the prevalence of *N. caninum* antibodies in goats was 3.9%, which is slightly higher than the 1.1% recorded locally [15], but lower than the seroprevalence of 26.4% reported in Brazil [42] and other Asian countries [43]. The low prevalence coincides with reports from Iran (6.2%) [44] and the Czech Republic (6.0%) [45]. Nevertheless, exposure to *N. caninum* should be considered a vital outcome as the parasite has been associated with deleterious effects on the feto-maternal structures and inhibitory action against post-parturient macrophage activities, which are required for normal fetal membrane release [46].

The presence of co-infection with any of the three protozoan parasites was also investigated in this study. Resultantly, only one animal was seropositive for *T. gondii* and *N. caninum* among the sampled cattle, and three were positive for *N. caninum* and *B. besnoiti*. These findings depict a low level of co-infection with any of the three parasites. In contrast, a study conducted on water buffaloes, which are considered large ruminants and the same category as cattle, revealed a 15.3% co-infection for both *T. gondii* and *N. caninum* [2].

Although the seroprevalence of co-infection of *T. gondii* and *N. caninum* was very low in goats (>2.0%), 5 of the 13 farms had at least one seropositive animal for both parasites. These findings suggest that the risk factors for both infections might be common on seropositive farms. Bartova et al. [45] employed the same cELISA for detecting anti-*N. caninum* antibodies and also found that all goats testing positive for *N. caninum* were also positive for *T. gondii*. The present study used an ELISA technique for the detection of both parasites, which is considered to have higher specificity and sensitivity compared to other serological methods [47]. Cross-reactions between *T. gondii* and *N. caninum* have been demonstrated to occur when IFAT was used given that a high concentration of fluorescent antibodies against apical organelles antigens of numerous apicomplexan parasites is common [48]. Notably, the majority of goats seropositive for *T. gondii* and *N. caninum* were adult animals. These results reflect that most of the seropositive goats were probably chronically infected by both parasites. Given the public health significance and the risk of zoonotic transmission of both parasites, preventive measures to mitigate the exposure of ruminants to *T. gondii* and *N. Caninum* are pertinent.

Apart from the seroprevalence results, risk factor analysis is essential to identify and implement effective control programs against the three protozoan parasites investigated in this study. For the sampled cattle, the odds of having a *T. gondii*-seropositive animal were higher in semi-intensive compared to intensive farms. In Malaysian ruminant farms, semi-intensive management systems provide opportunities for livestock to graze externally in contrast to intensive systems where the animals are confined. Such practices heighten the exposure to oocysts, which are widely distributed in the environment, thereby increasing the risk of *T. gondii* infection [48].

For the goat population in this study, higher seroprevalence to *T. gondii* was observed among older animals (>12 months), semi-intensively managed farms, as well as those with larger herd sizes, presence of cats or dogs, and using single-source/own animals as replacement stock. Recent seroprevalence studies in goats have also demonstrated positive associations between seropositivity for *T. gondii* antibodies and increasing age [2], the rearing system used, and the presence of dogs and cats [7]. The association between increasing age and a higher *T. gondii* infection suggests sustained exposure to the parasite and the situation could become exacerbated due to a weakening immune system. Such animals are more likely to be exposed to these infections from various sources since they have lived longer. Therefore, older goats are less resistant to *T. gondii* infection as a result of lower defense mechanisms associated with aging [3,48].

On the other hand, cats are the definitive host for *T. gondii*, and a single infected cat sheds millions of oocysts which allows them to rapidly contaminate a wide area. Moreover, in most of the visited farms in this study, cats and dogs had access to water troughs and animal feeds that were kept outdoors. These events might heighten the seropositivity for goats included from the various farms. A study conducted in Klang Valley, Malaysia by Tan [49] detected that 10.5% of cats sampled shed *T. gondii* DNA in their feces while 5.5% were seropositive against *T. gondii* antibodies, indicating a significant threat to farming, as well as representing a public health hazard. As also reported in a recent review [50], the presence of cats on farms and their movements in housing or feed storage areas increase the risk of *T. gondii* infection. The significant role of cats in the maintenance and transmission of caprine toxoplasmosis has been demonstrated in previous research [51]. Hence, animal sheds or farms should be subjected to good hygienic practices.

The limitations in this study are attributed to the study design, diagnostic method used, and study location. Given that this research entailed a cross-sectional design, the findings only reflect snap-shot information of exposure levels to the three apicomplexan parasites investigated among the studied population. Causal relationships could not be obtained as only associations between potential risk factors and seropositivity to *T. gondii* antibodies were analyzed. The prevalence of *N. caninum* and *B. besnoiti* was too low to accommodate risk factor analysis, hence future studies might consider enrolling a larger sample size to facilitate such analysis. The indirect diagnosis of the three protozoans by ELISA tests may impact some level of cross-reactivity [3]. Notwithstanding, the three commercial ELISA kits employed in this study were specific for multiple species of small and large ruminants, with high sensitivity and specificity values as reported in previous research [14]. Since only ruminant farms located in Selangor were considered in the present study, similar investigations are required to elucidate the information in other states in Malaysia.

## 5. Conclusions

In conclusion, this study suggests that toxoplasmosis is highly prevalent among the goat population in farms located in Selangor, Malaysia, whereas *N. caninum* infections were relatively low. Overall, sampled cattle from the studied area demonstrated an overall low exposure to the three protozoan parasites. The semi-intensive management of cattle farms was identified as the main risk factor for bovine toxoplasmosis, whereas multiple factors such as older animals, a larger herd size, the presence of cats or dogs, and using single source/own animals as replacement stock were associated with a higher prevalence of *T. gondii* in goats. Given the vital role of these parasites in causing reproduction disorders, controlling the infections is pertinent for economic, animal welfare, and public health reasons. These findings are vital for veterinarians, animal, and human health bodies to strategize effective control measures against these parasites in Malaysia. More national epidemiological research is needed to succinctly elucidate the spatial distribution of these infections and estimate the potential impact on Malaysia’s livestock industry.

## Figures and Tables

**Table 1:** Farm and animal characteristics of the sampled animals from ruminant farms in Selangor, Malaysia.

	Cattle (n = 225)		Goats (n = 179)		Overall (n = 404)	
Variables	Frequency	Percentage	Frequency	Percentage	Frequency	Percentage
**Age (months)**						
** 1–12 **	44	19.6	49	27.4	93	23.0
** 13–24 **	83	36.9	90	50.3	173	42.8
** Above 24 **	98	43.6	40	22.3	138	34.1
**Sex**						
** Male **	16	7.1	27	15.1	43	10.6
** Female **	209	92.9	152	84.9	361	89.4
**Management**						
** Intensive **	193	85.8	139	77.7	332	82.1
** Semi-intensive **	32	14.2	40	22.3	72	17.9
**Pest control**						
** Yes **	47	20.9	36	20.1	83	20.5
** No **	178	79.1	143	79.9	321	79.5
**Presence of dogs, cats, and wild animals**						
** Yes **	131	58.2	137	76.9	268	66.3
** No **	94	41.8	42	23.5	136	33.7
**Animal source**						
** Single/internal **	124	55.1	84	46.9	208	51.4
**Multiple/external**	101	44.9	95	53.1	196	48.6
**Herd size**						
** Less than 50 **	77	34.2	45	25.1	122	30.1
** 50–99 **	30	13.3	54	30.2	84	20.7
** 100 and above **	118	52.4	80	44.7	198	49.0
**Main production**						
** Meat **	24	10.7	127	70.9	151	37.4
** Milk **	126	56.0	30	16.8	156	38.6
** NA **	75	23.3	22	12.3	97	24.0
**Water source**						
** Tap water **	147	65.3	145	81.0	292	72.3
** Tap water + underground **	6	2.7	10	5.6	16	3.9
** Underground **	9	4.0	8	4.5	17	4.2
** Public source **	55	24.4	16	8.9	71	17.5
** Well **	8	3.6	-		8	1.9

NA = not available.

**Table 2:** Seroprevalence of *T. gondii*, *N. caninum*, and *B. besnoiti* antibodies in cattle (n = 225) sampled from 19 farms in Selangor, Malaysia.

Farms Label	Number of Sampled Animals	Seropositivity for *T. gondii* (%)	Seropositivity for *N. caninum* (%)	Seropositivity for *B. besnoiti* (%)
**F1**	10	0 (0.0)	1 (10.0)	0 (0.0)
**F2**	5	1 (20.0)	0 (0.0)	1 (20.0)
**F3**	2	0 (0.0)	0 (0.0)	1 (50.0)
**F4**	9	0 (0.0)	0 (0.0)	5 (55.6)
**F5**	9	4 (44.4)	1 (11.1)	1 (11.1)
**F6**	4	0 (0.)	0 (0.0)	0 (0.0)
**F7**	5	0 (0.0)	0 (0.0)	0 (0.0)
**F8**	8	1 (12.5)	0 (0.0)	0 (0.0)
**F9**	9	0 (0.0)	0 (0.0)	0 (0.0)
**F10**	8	0 (0.0)	0 (0.0)	1 (12.5)
**F11**	16	0 (0.0)	1 (6.3)	0 (0.0)
**F12**	37	0 (0.0)	3 (8.1)	4 (10.8)
**F13**	13	0 (0.0)	0 (0.0)	0 (0.0)
**F14**	12	1 (8.3)	0 (0.0)	0 (0.0)
**F15**	2	1 (50.0)	0 (0.0)	0 (0.0)
**F16**	10	0 (0.0)	0 (0.0)	0 (0.0)
**F17**	30	1 (3.3)	0 (0.0)	0 (0.0)
**F18**	30	3 (10.0)	0 (0.0)	0 (0.0)
**F19**	6	0 (0.0)	0 (0.0)	0 (0.0)
**Total**	**225**	**12 (5.3)**	**6 (2.7)**	**13 (5.7)**

**Table 3:** Seroprevalence of *T. gondii*, *N. caninum*, and *B. besnoiti* antibodies in goats (n = 179) sampled from 13 farms in Selangor, Malaysia.

Farms Label	Number of Sampled Animals	Seropositivity for *T. gondii* (%)	Seropositivity for *N. caninum* (%)
**F1**	20	5 (20.0)	2 (10.0)
**F2**	30	14 (30.0)	0 (0.0)
**F3**	10	10 (100.0)	0 (0.0)
**F4**	15	13 (86.7)	0 (0.0)
**F5**	6	6 (100.0)	1 (16.7)
**F6**	10	9 (90.0)	1 (10.0)
**F7**	11	10 (90.9)	2 (18.2)
**F8**	5	0 (0.0)	0 (0.0)
**F9**	8	6 (75.0)	1 (12.5)
**F10**	18	15 (83.3)	0 (0.0)
**F11**	24	22 (91.7)	0 (0.0)
**F12**	16	13 (81.3)	0 (0.0)
**F13**	6	2 (33.3)	0 (0.0)
**Overall**	**179**	**125 (69.8)**	**7 (3.9)**

**Table 4:** Final mixed-effects logistic regression model of the factors associated with the prevalence of *T. gondii* antibodies in goats from 13 farms in Selangor, Malaysia.

Variables	B	S.E.	Wald	df	OR	95% CI	*p*-Value
**Age (months)**			8.51	2			0.014
Above 12	−1.63	0.56	8.43	1	5.26	1.7–16.66	0.004
7–12	−1.01	0.56	3.23	1	2.77	0.91–8.33	0.072
Less than 6					Ref		
**Management**							
**Intensive**	−0.76	0.49	2.43	1	2.17	1.34–6.21	0.12
Semi-intensive					Ref		
**Presence of dogs, cats, and wild animals**							
Yes	1.29	0.62	4.28	1	3.63	1.1–12.3	0.03
No					Ref		
**Animal source**							
Single/internal	1.37	0.45	9.09	1	3.92	1.61–9.69	0.00
Multiple/external					Ref		
**Herd size**			6.84	2			0.00
100 and above	−1.30	0.50	6.83	1	3.70	1.38–10.0	0.00
50–99	−0.50	0.49	1.05	1	1.67	0.63–4.34	0.30
Less than 50					Ref		

Note: df = degree of freedom, CI = confidence interval, OR = odds ratio, ref = reference group.

**Table 5:** Final mixed-effects logistic regression model of the factors associated with the prevalence of *T. gondii* antibodies in cattle from 16 farms in Selangor, Malaysia.

Variables	B	S.E.	Wald	df	OR	95% CI	*p*-Value
**Management**							
** Intensive **	−1.59	0.62	6.59	1	0.20	0.06–0.68	0.01
** Semi-intensive **					Ref		
**Presence of dogs, cats, and wild animals**							
** Yes **	1.60	1.12	2.04	1	4.99	0.55–45.35	0.15
** No **					Ref		
**Pest control**							
** Yes **	−2.00	1.39	2.07	1	0.13	0.009–2.06	0.15
** No **					Ref		

Note: df = degree of freedom, CI = confidence interval, OR = odds ratio, ref = reference group.

## Data Availability

Data will be made available upon request.

## References

[1] Waap H., Bärwald A., Nunes T., Schares G. (2022). Seroprevalenceand Risk Factors for *Toxoplasma gondii* and *Neospora caninum* in Cattle in Portugal. Animals.

[2] Ciuca L., Borriello G., Bosco A., D’Andrea L., Cringoli G., Ciaramella P., Maurelli M.P., Di Loria A., Rinaldi L., Guccione J. (2020). Seroprevalence and clinical outcomes of neospora caninum, toxoplasma gondii, and besnoitia besnoiti infections in water buffaloes(Bubalus bubalis). Animals.

[3] Ali S., Amjad Z., Khan T.M., Maalik A., Iftikhar A., Khan I., Ahmed H. (2020). Occurrence of *Toxoplasma gondii* antibodies and associated risk factors in women in selected districts of Punjab province, Pakistan. Parasitology.

[4] Dubey J.P., Murata F.H.A., Cerqueira-Cézar C.K., Kwok O.C.H., Yang Y.R. (2020). Public Health Significance of Toxoplasma gondii Infections in Cattle: 2009–2020. J. Parasitol..

[5] Garcia-Bocanegra I.G., Cabezòn O., Hernandez E., Martinez-Cruz M.S., Martinez-Moreno A., Martinez-Moreno J. (2013). *Toxoplasma gondii* in ruminant species (cattle, sheep, and goats) from Southern Spain. J. Parasitol..

[6] Fowler C. (2017). New Study Estimates Toxoplasmosis Costs Sheep Industry $70 Million per Year in South Australia. https://ab.co/2Mgd5px.

[7] Gazzonis A.L., Alvarez Garcia G., Zanzani S.A., Ortega Mora L.M., Invernizzi A., Manfredi M.T. (2016). Neospora caninum infection in sheep and goats from north-eastern Italy and associated risk factors. Small Rumin. Res..

[8] Tzanidakis N., Maksimov P., Conraths F.J., Kiossis E., Brozos C., Sotiraki S., Schares G. (2012). Toxoplasma gondii in sheep and goats: Seroprevalence and potential risk factors under dairy husbandry practices. Vet. Parasitol..

[9] Cortes H., Leitão A., Gottstein B., Hemphill A. (2014). A review on bovine besnoitiosis: A disease with economic impact in herd health management, caused by Besnoitia besnoiti (Franco and Borges). Parasitology.

[10] Jacquiet P., Liénard E., Franc M. (2010). Bovine besnoitiosis: Epidemiological and clinical aspects. Vet. Parasitol..

[11] Álvarez-García G., Fernández-García A., Gutiérrez-Expósito D., Quiteria J.A.R.S., Aguado-Martínez A., Ortega-Mora L.M. (2014). Seroprevalence of Besnoitia besnoiti infection and associated risk factors in cattle from an endemic region in Europe. Vet. J..

[12] Rostaher A., Mueller R.S., Majzoub M., Schares G., Gollnick N.S. (2010). Bovine besnoitiosis in Germany. Vet. Dermatol..

[13] Fernandez-Garcia A., Risco-Castillo V., Pedraza-Diaz S., Aguado-Martinez A., Alvarez-Garcia G., Gomez-Bautista M., Collantes-Fernandez E., Ortega-Mora L.M. (2009). First isolation of Besnoitia besnoiti from a chronically infected cow in Spain. J. Parasitol..

[14] Gollnick N.S., Scharr J.C., Schares G., Langenmayer M.C. (2015). Natural Besnoitia besnoiti infections in cattle: Chronology of disease progression. BMC Vet. Res..

[15] Abdul Hamid N., Sadiq M.B., Ramanoon S.Z., Mansor R., Watanabe M., Md Isa N.M., Kamaludeen J., Syed-Hussain S.S. (2020). Seroprevalence and risk factors of *Toxoplasma gondii* in ruminant meats from wet markets in Klang valley and abattoirs in Selangor, Malaysia. Animals.

[16] Watanabe M., Sadiq M.B., Mulop N.I.A., Mohammed K., Rani P.A.M., Fong L.S., Aziz N.A., Kamaludeen J., Ramanoon S.Z., Mansor R. (2020). Prevalence of *Toxoplasma gondii* antibodies in stray dogs from various locations in west and east Malaysia. Korean J. Parasitol..

[17] Kyaw T., Athirah M.M., Kalthum H., Abd Rahman A., Aklilu E., Hoe C.H., Chandrawathani P. *Neospora caninum* antibody detection in sheep and goats in Kelantan. Proceedings of the 52nd Annual Scientific Conference, Malaysian Society of Parasitology and Tropical Medicine, Grand Season Hotel.

[18] Cheah T.S., Mattsson J.G., Zaini M., Sani R.A., Jakubek E.B., Uggla A., Chandrawathani P. (2004). Isolation of *Neospora caninum* from a calf in Malaysia. Vet. Parasitol..

[19] Rahman W., Manimegalai V., Chandrawathani P., Nurulaini R., Zaini C., Premaalatha B. (2011). Seroprevalence of Toxoplasma gondii in Malaysian cattle. Malays. J. Vet. Res..

[20] Chandrawathani P., Nurulaini R., Zanin C.M., Premaalatha B., Adnan M., Jamnah O., Zatil S.A. (2008). Seroprevalence of Toxoplasma gondii antibodies in pigs, goats, cattle, dogs, and cats in peninsular Malaysia. Trop. Biomed..

[21] Zayadi R.A. (2019). Current outlook of the Livestock Industry in Malaysia and ways toward sustainability. J. Sus. Nat. Res..

[22] Von Blumröder D., Schares G., Norton R., Williams D.J.L., Esteban-Redondo I., Wright S., Björkman C., Frössling J., Risco-Castillo V., Fernández-García A. (2004). Comparison and standardization of serological methods for the diagnosis of *Neospora caninum* infection in bovines. Vet. Parasitol..

[23] García-Lunar P., Ortega-Mora L.M., Schares G., Gollnick N.S., Jacquiet P., Grisez C., Prevot F., Frey C.F., Gottstein B., Álvarez-García G. (2013). An inter-laboratory comparative study of serological tools employed in the diagnosis of *Besnoitia besnoiti* infection in bovines. Transbound. Emerg. Dis..

[24] Shariatzadeh S.A., Sarvi S., Hosseini S.A., Sharif M., Gholami S., Pagheh A.S., Montazeri F., Nayeri T., Nakhaei M., Mikaeili Galeh T. (2021). The global seroprevalence of *Toxoplasma gondii* infection in bovines: A systematic review and meta-analysis. Parasitology.

[25] Al-Kappany Y.M., Abbas I.E., Devleesschauwer B., Dorny P., Jennes M., Cox E. (2018). Seroprevalence of anti-*Toxoplasma gondii* antibodies in Egyptian sheep and goats. BMC Vet. Res..

[26] Bahrami S., Zarei M., Ghorbanpour M., Karami S. (2000). *Toxoplasma gondii* in sheep and goat livers: Risks for human consumption. J. Hell. Vet. Med. Soc..

[27] Ahmed H., Malik A., Arshad M., Mustafa I., Khan M.R., Afzal M.S., Simsek S. (2016). Seroprevalence and spatial distribution of toxoplasmosis in sheep and goats in North-Eastern region of Pakistan. Korean J. Parasitol..

[28] Bawm S., Maung W.Y., Win M.Y., Thu M.J., Chel H.M., Khaing T.A., Tiwananthagorn S. (2016). Serological survey and factors associated with *Toxoplasma gondii* infection in domestic goats in Myanmar. Scientifica.

[29] Amdouni Y., Rjeibi M.R., Rouatbi M., Amairia S., Awadi S., Gharbi M. (2017). Molecular detection of Toxoplasma gondii infection in slaughtered ruminants (sheep, goats, and cattle) in Northwest Tunisia. Meat Sci..

[30] Özdal N., Oğuz B., Orunç Kılınç Ö., Karakuş A., Değer S. (2019). Prevalence of ELISA-detected specific antibodies against Besnoitia besnoiti in cattle of the Eastern and Southeastern Anatolian regions, Turkey. Iran. J. Vet. Res..

[31] Nasir A., Lanyon S.R., Schares G., Anderson M.L., Reichel M.P. (2012). Sero-prevalence of Neospora caninum and Besnoitia besnoiti in South Australian beef and dairy cattle. Vet. Parasitol..

[32] Papadopoulos E., Arsenos G., Ptochos S., Katsoulos P., Oikonomou G., Karatzia M.A., Karatzias H. (2014). First report of Besnoitiabesnoiti seropositive cattle in Greece. J. Hell. Vet. Med. Soc..

[33] Rinaldi L., Maurelli M.P., Musella V., Bosco A., Cortes H., Cringoli G. (2013). First cross-sectional serological survey on Besnoitia besnoiti in cattle in Italy. Parasitol. Res..

[34] Esteban-Gil A., Calvete C., Casasús I., Sanz A., Ferrer J., Peris M.P., Marcén-Seral J.M., Castillo J.A. (2017). Epidemiological patterns of bovine besnoitiosis in an endemic beef cattle herd reared under extensive conditions. Vet. Parasitol..

[35] Schares G., Langenmayer M.C., Scharr J.C., Minke L., Maksimov P., Maksimov A., Gollnick N.S. (2013). Novel tools for the diagnosis and differentiation of acute and chronic bovine besnoitiosis. Int. J. Parasitol..

[36] Australian Malaysia—Country Commercial Guide: Agricultural Sector. https://www.trade.gov/country-commercial-guides/malaysia-agricultural-sector.

[37] Stoessel Z., Taylor L.F., McGowan M.R., Coleman G.T., Landmann J.K. (2003). Prevalence of antibodies to Neospora caninum within central Queensland beef cattle. Aust. Vet. J..

[38] Liénard E., Salem A., Jacquiet P., Grisez C., Prévot F., Blanchard B., Franc M. (2013). Development of a protocol testing the ability of Stomoxys calcitrans (Linnaeus, 1758) (Diptera: Muscidae) to transmit Besnoitia besnoiti (Henry, 1913) (Apicomplexa: Sarcocystidae). Parasitol. Res..

[39] HeoChong C., Ahmad N.W., ChewWai K., Kurahashi H., Jeffery J., HeahSock K., Omar B. (2010). A study of cow dung Diptera in Sentul Timur, Kuala Lumpur, Malaysia. J. Trop. Med. Parasitol..

[40] Kyaw T., Mohd Mokhtar A., Ong B.L., Hoe C.H., Aziz A.R., Aklilu E., Kamarudin S. (2018). Seroprevalence of *Neospora Caninum* in Sheep and Goats of Gua Musang District in Kelantan, Malaysia. Pertanika J. Trop. Agric. Sci..

[41] Reichel M.P., Alejandra Ayanegui-Alcerreca M., Gondim L.F., Ellis J.T. (2013). What is the global economic impact of *Neospora caninum* in cattle—The billion dollar question. Int. J. Parasitol..

[42] Rodrigues A.A., Reis S.S., da Silva Moraes E., do Nascimento Souza Filho J.G., dos Santos Reis M.H., Martins T.A. (2021). Sero-prevalence and risk factors for Neospora caninum and Toxoplasma gondii in goats of Maranhão State. Brazil Vet. Parasitol..

[43] Ahmad S., and Tasawar Z. (2016). Seroprevalence of Toxoplasmosis in Small Ruminants from Cholistan Desert and Agricultural Areas of Rahim Yar Khan and Rajan Pur (Punjab) Pakistan. Pakistan J. Zool..

[44] Gharekhani J., Esmaeilnejad B., Rezaei H., Yakhchali M., Heidari H., Azhari M. (2016). Prevalence of anti-Neospora caninum antibodies in Iranian goats. Ann. Parasitol..

[45] Bartova E., Sedlak K. (2012). *Toxoplasma gondii* and *Neospora caninum* antibodies in goats in the Czech Republic. Vet. Med..

[46] Sánchez-Sánchez R., Vázquez-Calvo Á., Fernández-Escobar M., Regidor-Cerrillo J., Benavides J., Gutiérrez J., Gutiérrez-Expósito D., Crespo-Ramos F.J., Ortega-Mora L.M., Álvarez-García G. (2021). Dynamics of neospora caninum-associated abortions in a dairy sheep flock and results of a test-and-cull control programme. Pathogens.

[47] Lindsay D.S., Dubey J.P. (2020). Neosporosis, toxoplasmosis, and sarcocystosis in ruminants: An update. Vet. Clin. North Am. Food Anim. Pract..

[48] Dubey J.P. (2010). Toxoplasmosis of Animals and Humans.

[49] Tan L.P., Megat Abd Rani P.A., Sharma R.S.K., Syed Hussain S.S., Watanabe M. (2020). Prevalence of Toxoplasma gondii in pet and stray cats in Klang Valley, Malaysia. Trop. Biomed..

[50] Stelzer S., Basso W., Benavides Silván J., Ortega-Mora L.M., Maksimov P., Gethmann J., Conraths F.J., Schares G. (2019). Toxoplasma gondii infection and toxoplasmosis in farm animals: Risk factors and economic impact. Food Waterborne Parasitol..

[51] Fortes M.S., Lopes-Mori F.M.R., Caldart E.T., Constantino C., Evers F., Pagliari S., de Almeida J.C., Barros L.D., Freire R.L., Garcia J.L. (2018). Caprine toxoplasmosis in Southern Brazil: A comparative seroepidemiological study between the indirect immunofluorescence assay, the enzyme-linked immunosorbent assay, and the modified agglutination test. Trop. Anim. Health Prod..

